# Predicting surgical outcomes in single-port robot-assisted partial nephrectomy: external validation and comparative analysis of PADUA, RENAL, and SPARE scores

**DOI:** 10.1007/s00345-025-06081-7

**Published:** 2025-11-20

**Authors:** Filippo Carletti, Fabio Maria Valenzi, Flavia Tamborino, Alexandru Turcan, Valerio Santarelli, Arianna Biasatti, Luca Alfredo Morgantini, Hakan Bahadir Haberal, Srinivas Vourganti, Fabrizio Dal Moro, Riccardo Autorino, Simone Crivellaro

**Affiliations:** 1 Department of Urology, University of Illinois at Chicago, Chicago, IL USA; 2 Urology Clinic, Department of Surgery, Oncology and Gastroenterology, University of Padua, Padua, Italy; 3 Department of Urology, Rush University Medical Center, Chicago, IL USA

**Keywords:** Single-port robot-assisted partial nephrectomy, SP-RAPN, PADUA, RENAL, SPARE, trifecta, nephrometry scores, SP, single port, outcomes, RAPN

## Abstract

**Purpose:**

Nephrometry scores are essential tools for classifying and comparing tumor complexity and guiding surgical planning in partial nephrectomy. However, their performance in single-port robot-assisted partial nephrectomy (SP-RAPN) has not been formally assessed. We aimed to externally validate and compare the predictive performance of the Preoperative Aspects and Dimensions Used for an Anatomical (PADUA), Radius-Exophytic/Endophytic-Nearness-Anterior/Posterior-Location (RENAL), and Simplified PADUA Renal (SPARE) nephrometry scores in patients undergoing SP-RAPN.

**Methods:**

We retrospectively reviewed 211 consecutive patients who underwent SP-RAPN for solitary ≤ cT2 renal tumors at two academic centers between 2019 and 2024. The primary endpoint was Trifecta achievement, defined as the simultaneous presence of negative surgical margins, absence of perioperative complications, and warm ischemia time ≤ 25 min. Discrimination and clinical utility of each nephrometry score were assessed using receiver operating characteristic (ROC) curve analysis and decision curve analysis (DCA). Multivariable logistic regression adjusted for relevant clinical covariates.

**Results:**

Trifecta was achieved in 50.7% of patients. The SPARE score demonstrated the highest discriminative performance (AUC 0.681), followed by PADUA (0.661) and RENAL (0.654), though these differences were not statistically significant. DCA showed overlapping net benefit curves, with SPARE offering marginally superior. Limitations include the retrospective design and underrepresentation of highly complex tumors.

**Conclusions:**

PADUA, RENAL, and SPARE scores show comparable performance in predicting Trifecta achievement in the setting of SP-RAPN. SPARE may perform slightly better, and it represents a more practical choice for routine preoperative assessment due to its ease of use.

**Supplementary Information:**

The online version contains supplementary material available at 10.1007/s00345-025-06081-7.

## Introduction

Robot-assisted partial nephrectomy (RAPN) has become widely adopted for the treatment of T1 renal tumors [1]. More recently, the introduction of the single port (SP) da Vinci platform has led to a paradigm shift in surgical access. In this context, the retroperitoneal approach has gained favor in SP-RAPN due to its direct access to the renal hilum without the need for bowel mobilization.

Accurate and reproducible anatomical scoring systems are essential for standardizing tumor assessments, guiding surgical planning, and comparing perioperative outcomes across studies. Among the most established nephrometry scores are the Radius-Exophytic/Endophytic-Nearness-Anterior/Posterior-Location [2] (RENAL) score and the Preoperative Aspects and Dimensions Used for an Anatomical [3] (PADUA) score, introduced in 2009, which quantify tumor location, size, and relationship to key intrarenal structures. These first-generation scores are the standard for reporting complexity and predicting morbidity [4] but are limited by moderate interobserver variability and relatively complex scoring frameworks. To address these shortcomings, 10 years later, the Simplified PADUA Renal score [5] (SPARE) was introduced to retain predictive accuracy while improving usability and reproducibility.

Although several external validations of the PADUA, RENAL, and SPARE scores exist, in open [6–9], laparoscopic [10, 11], and multiport robotic settings [12–14], no large-scale study to date has specifically focused on the predictive performance of these scores in SP-RAPN. For this reason, the objective of our study is to provide a dedicated validation of these nephrometry scores within a SP-RAPN cohort.

## Materials and methods

### Study design

We retrospectively reviewed all patients who underwent SP-RAPN at the at two large academic institutions (University of Illinois at Chicago and Rush University Medical Center) between January 1, 2019, and December 31, 2024, for solitary ≤ cT2 renal tumors. All procedures were performed using the da Vinci SP system through either a retroperitoneal or transperitoneal approach. The decision between a traditional enucleoresection or tumor enucleation was made at the surgeon’s preference. Additional inclusion criteria were: (i) a computed tomography (CT) or magnetic resonance imaging (MRI) performed within 90 days prior to surgery with images available for review, and (ii) absence of lymph‑node or distant metastases (N0M0). Patients were excluded if they had a solitary kidney (*n* = 3), end‑stage renal disease (*n* = 4), recurrent renal cell carcinoma (*n* = 2), inadequate follow‑up (*n* = 8), or unavailable imaging in the institutional picture archiving and communication system (PACS) (*n* = 11), resulting in a final cohort of 211 patients with complete clinical and imaging data.

### Endpoints

The primary endpoint was to evaluate which nephrometry score best predicts surgical outcomes, assessed by the achievement of Trifecta following SP-RAPN. Trifecta [15] was defined as the simultaneous achievement of negative surgical margins, absence of perioperative complications, and warm ischemia time (WIT) ≤ 25 min. Secondary endpoints included the association between individual anatomical tumor features and Trifecta achievement.

### Variables

Demographics and comorbidities were recorded for all patients. Preoperative CT or MRI images were independently reviewed by two urologists blinded to all clinical information. After jointly evaluating the first 25 tumors to harmonise definitions, one rater assigned PADUA, RENAL, and SPARE scores (Supplementary Table 1) for all subsequent cases, while the second rater reviewed only those flagged as borderline. Ambiguous cases were resolved by consensus. Intraoperative data variables included surgical approach, estimated blood loss, operative time, WIT, use of vascular clamping, renorrhaphy technique, and conversion to open procedure or radical nephrectomy. Peri‑operative metrics comprised length of hospital stay and 90‑day complications, which were graded with the Clavien–Dindo classification. Pathology variables covered tumor histology and surgical margin status. Trifecta achievement was calculated according to established criteria [15].

### Statistical methods

Statistical analyses were performed with R software (version 4.4.1; R Foundation for Statistical Computing, Vienna, Austria). Two‑sided p‑values < 0.05 were considered statistically significant. Continuous variables were reported as median (interquartile range [IQR]) and compared with Mann–Whitney U or Kruskal–Wallis tests; categorical variables were expressed as number (percentage) and compared with χ² or Fisher’s exact tests. Univariate and multivariable logistic regression models were used to evaluate factors associated with the achievement of Trifecta. Three separate models were constructed in which RENAL, PADUA, and SPARE scores were entered individually as the main predictors and adjusted for age, body mass index (BMI), Charlson Comorbidity Index (CCI), and tumor size. The discriminative ability of each nephrometry score was quantified using the area under the curve (AUC) of the receiver operating characteristic (ROC) curve with 95% confidence intervals. Pairwise AUC comparisons were performed using DeLong’s test. Decision curve analysis [16] (DCA) was employed to assess the net clinical benefit of each score across threshold probabilities from 5% to 30%. Component‑level analyses were performed by entering the individual items of each nephrometry system into separate univariate and multivariable logistic regression models to identify which anatomical features drive the predictive performance of Trifecta achievement.

## Results

### Baseline characteristics

Baseline patient and tumor characteristics are summarized in Table 1. A total of 211 patients met the inclusion criteria. The median age was 60 years (IQR: 51–67), the majority were male (54%), with a median BMI of 30.1 kg/m² (IQR: 26.1–35.4). Hypertension (69%), hypercholesterolemia (39%), and diabetes mellitus (29%) were the most prevalent comorbidities, 63.5% were classified as American Society of Anesthesiologists (ASA) score III-IV, and 39.8% had a history of previous abdominal surgery. Median estimated glomerular filtration rate decreased slightly from 80.4 to 78.5 mL/min/1.73 m².

**Table 1:** Baseline characteristics

Characteristics		*N* = 211
Patient	Age, median (IQR)	60 (51–67)
	Male, n (%)	114 (54)
	Race, n (%) White African American Hispanic Asian/Pacific Indian Other	80 (37.9) 71 (33.6) 32 (15.2) 8 (3.8) 19 (9.0) 1 (0.5)
	BMI (kg/m²), median (IQR)	30.1 (26-35.4)
	ASA, n (%) 1 2 3 4	5 (2.4) 72 (34.1) 131 (62.1) 3 (1.4)
	Charlson Comorbidity Index, median (IQR)	3 (2–4)
	Hypertension, n (%)	145 (68.7)
	Hypercholesterolemia, n (%)	83 (39.3)
	Diabetes, n (%)	61 (28.9)
	Obesity, n (%)	79 (37.4)
	Prior abdominal surgery, n (%)	84 (39.8)
	Preoperative eGFR, (mL/min/1.73 m^2^),median (IQR)	80.4 (58.3–95.4)
Tumor	Right Side, n (%)	113 (53.6)
	Tumor size, (cm), median (IQR)	3.0 (2.3-4.0)
	Clinical T, n (%) 1(T1a) 2(T1b) 3(T2a)	166 (78.7) 39 (18.5) 6 (2.8)
	Renal mass location, n (%) Upper pole Interpolar Lower pole	67 (31.8) 55 (26) 89 (42.2)
	PADUA score, median (IQR)	7 (7–9)
	PADUA risk group, n (%) Low Intermediate High	42 (19.9) 138 (65.4) 31 (14.7)
	SPARE score, median (IQR)	2 (0–3)
	SPARE risk group, n (%) Low Intermediate High	165 (78.2) 36 (17.1) 10 (4.7)
	RENAL score, median (IQR)	6 (5–7)
	RENAL risk group, n (%) Low Intermediate High	142 (67.3) 57 (27.0) 12 (5.7)

*ASA*  American Society of Anesthesiologists, *BMI * body mass index, *CCI *charlson comorbidity index, *eGFR * estimated glomerular filtration rate, *IQR *Interquartile Range, *PADUA * preoperative Aspects and Dimensions Used for an Anatomical classification, *SPARE* simplified PADUA REnal nephrometry score, *RENAL * radius , Exophytic/endophytic, nearness to collecting system/sinus, anterior/posterior, location relative to polar lines.

Tumors were more frequently located on the right side (53.6%) and in the lower pole (42.2%), followed by the upper pole (31.8%) and the interpolar region (26.1%). The median tumor size was 3.0 cm (IQR: 2.3–4.0 cm).

Detailed anatomical components contributing to each nephrometry score are reported in Supplementary Table 2. Nephrometry scores showed a median PADUA score of 7 (IQR: 7–9), SPARE score of 2 (IQR: 0–3), and RENAL score of 6 (IQR: 5–7). Based on these scores, most tumors were classified as intermediate complexity by PADUA (65.4%), low risk by SPARE (78.2%), and low complexity by RENAL (67.3%).

### Surgical outcomes

Surgical outcomes are summarized in Table 2. Most procedures were performed with a retroperitoneal approach (83.9%), using either lateral flank incision (56.9%) or low anterior access (43.1%). Enucleoresection was performed in 56.9% of cases, while pure enucleation was performed in 43.1%. The median operative time was 215 min (IQR: 158.5–256.5), and the median estimated blood loss was 100 mL (IQR: 50–200). Traditional clamping was applied in 83.4% of cases, with a median WIT of 22 min (IQR: 18–30). Intraoperative complications occurred in 7 patients (3.3%), including one conversion to radical nephrectomy and two conversions to open partial nephrectomy. Postoperative complications occurred in 21 patients (9.9%), including 14 patients (6.6%) requiring readmission and 7 events graded Clavien Dindo ≥ 3. The median length of hospital stay was 27 h (IQR 10–33). At final pathology, most tumors were classified as pT1a (78.7%), and 80.6% were confirmed malignant. Trifecta was achieved in 50.71% of cases.

**Table 2:** Surgical outcomes

**Approach, n (%)**	
Retroperitoneal	177 (83.9)
Transperitoneal	34 (16.1)
**Access, n (%)**	
Flank position	120 (56.9)
Low anterior access	91 (43.1)
**Technique, n (%)**	
Enucleoresection	120 (56.8)
Pure Enucleation	91 (43.1)
Operative time, median (IQR)	215 (158.5-256.5)
Estimated Blood Loss, median (IQR)	100 (50-200)
Off clamping, n (%)	35 (16.6)
Postoperative eGFR, (mL/min/1.73 m^2^),median (IQR)	78.5 (56.1-93.6)
Intraoperative complication, n (%)	7 (3.3)
**Postoperative complications, n (%)**	21 (9.9)
Grade 1	9 (42.9)
Grade 2	5 (23.8)
Grade 3	5 (23.8)
Grade 4	2 (9.5)
Length of Hospital Stay, hours, median (IQR)	27 (10-33)
Readmission, n (%)	14 (6.6)
**Histology, n (%)**	
Malignant	170 (80.6)
Benign	41 (19.4)
Positive Surgical Margins, n (%)	16 (7.6)
Trifecta achievement, n (%)	107 (50.7)

*IQR* interquartile range, *eGFR* estimated glomerular filtration rate.

Among the 104 patients who did not achieve Trifecta, the most frequent limiting factor was prolonged ischemia time, observed in 68 patients. The median WIT in this subgroup was 30 min (IQR: 25.5–36.5). Perioperative complications contributed to 28 failures, while positive surgical margins were present in 16 patients.

### Predictors of trifecta achievement

Multivariate analysis (Table 3) demonstrated that the PADUA and SPARE scores were significantly associated with Trifecta achievement in both intermediate vs. low risk groups (PADUA, OR: 0.33; 95% CI: 0.146–0.722; *p* = 0.007), (SPARE, OR: 0.36; 95% CI: 0.157–0.796; *p* = 0.013) and high vs. low risk groups (PADUA, OR: 0.11; 95% CI: 0.036–0.327; *p* < 0.001), (SPARE, OR: 0.18; 95% CI: 0.027–0.825; *p* = 0.045). The RENAL score was significantly associated with Trifecta achievement only in the intermediate risk group (OR 0.46; 95% CI: 0.233–0.899; *p* = 0.025). Longer operative time was significantly associated with failure to achieve Trifecta (OR :0.992; *p* < 0.001). Other covariates were non‑significant. In both univariate and multivariate item-level analyses (Supplementary Table 3) the presence of an exophytic component < 50%, a predominantly endophytic growth pattern, and tumor size of 4.1–7 cm were significantly associated with failure to achieve Trifecta across all scoring systems. In contrast, tumor location (anterior vs. posterior or upper/lower pole vs. interpolar) showed no association.

**Table 3:** Logistic regression predicting trifecta

	Univariate		Multivariate					
			PADUA		RENAL		SPARE	
Variables	OR (95%CI)	*P* value	OR (95%CI)	*P* value	OR (95%CI)	*P* value	OR (95%CI)	*P* value
Female vs. male	1.239 (0.721–2.137	0.438	1.126 (0.593–2.142)	0.716	1.114 (0.592–2.101)	0.736	1.062 (0.565–1.996)	0.850
Retroperitoneal vs. transperitoneale	1.829 (0.872–3.967)	0.115	1.301 (0.556–3.099)	0.546	1.140 (0.499–2.638)	0.756	1.245 (0.543–2.886)	0.604
Charlson comorbidity index	1.037 (0.911–1.821)	0.583	1.074 (0.931–1.244)	0.329	1.069 (0.929–1.233)	0.356	1.081 (0.938–1.249)	0.285
Prior abdominal surgery	1.209 (0.697–2.107)	0.499	1.040 (0.545–1.983)	0.905	1.053 (0.550–2.012)	0.876	1.114 (0.593–2.096)	0.736
Operative time	0.992 (0.988–0.996)	**< 0.001**	0.992 (0.987–0.996)	**< 0.001**	0.992 (0.987–0.996)	**< 0.001**	0.992 (0.988–0.997)	**0.001**
**PADUA score groups**								
Intermediate vs. low	0.335 (0.150–0.701)	**0.005**	0.334 (0.146–0.722)	**0.007**				
High vs. low	0.145 (0.049–0.396)	**< 0.001**	0.115 (0.036–0.327)	**< 0.001**				
**RENAL score groups**								
Intermediate vs. low	0.473 (0.249–0.882)	**0.019**			0.462 (0.233–0.899)	**0.025**		
High vs. low	0.377 (0.097–1.253)	0.124			0.297 (0.072–1.042)	0.068		
**SPARE score groups**								
Intermediate vs. low	0.387 (0.176–0.812)	**0.014**					0.363 (0.157–0.796)	**0.013**
High vs. low	0.194 (0.029–0.801)	**0.042**					0.189 (0.027–0.825)	**0.045**

*OR * odds ratio, *CI * confidence interval, *PADUA* preoperative aspects and dimensions used for an anatomical classification, *RENAL * radius exophytic/endophytic, nearness, anterior/posterior, location, *SPARE* simplified PADUA renal nephrometry score.

**Fig. 1:**
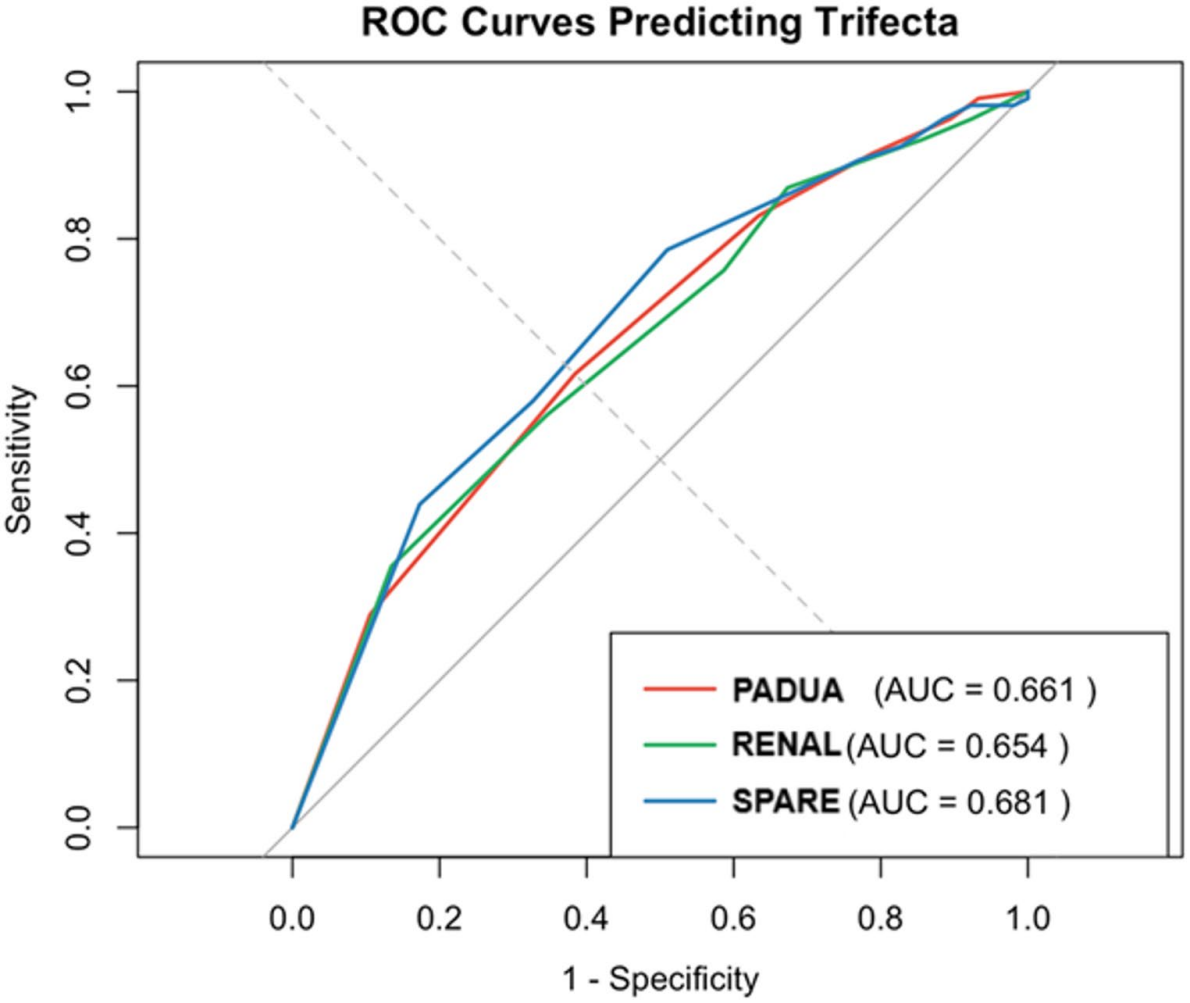
ROC curves predicting Trifecta

ROC analysis confirmed that the SPARE score provided the highest discrimination for Trifecta achievement (Fig. 1). SPARE reached an AUC of 0.681, exceeding PADUA (0.661) and RENAL (0.654). Pairwise DeLong comparisons did not reach statistical significance, with p‑values of 0.733 (PADUA vs. RENAL), 0.29 (PADUA vs. SPARE), and 0.357 (RENAL vs. SPARE).

**Fig. 2:**
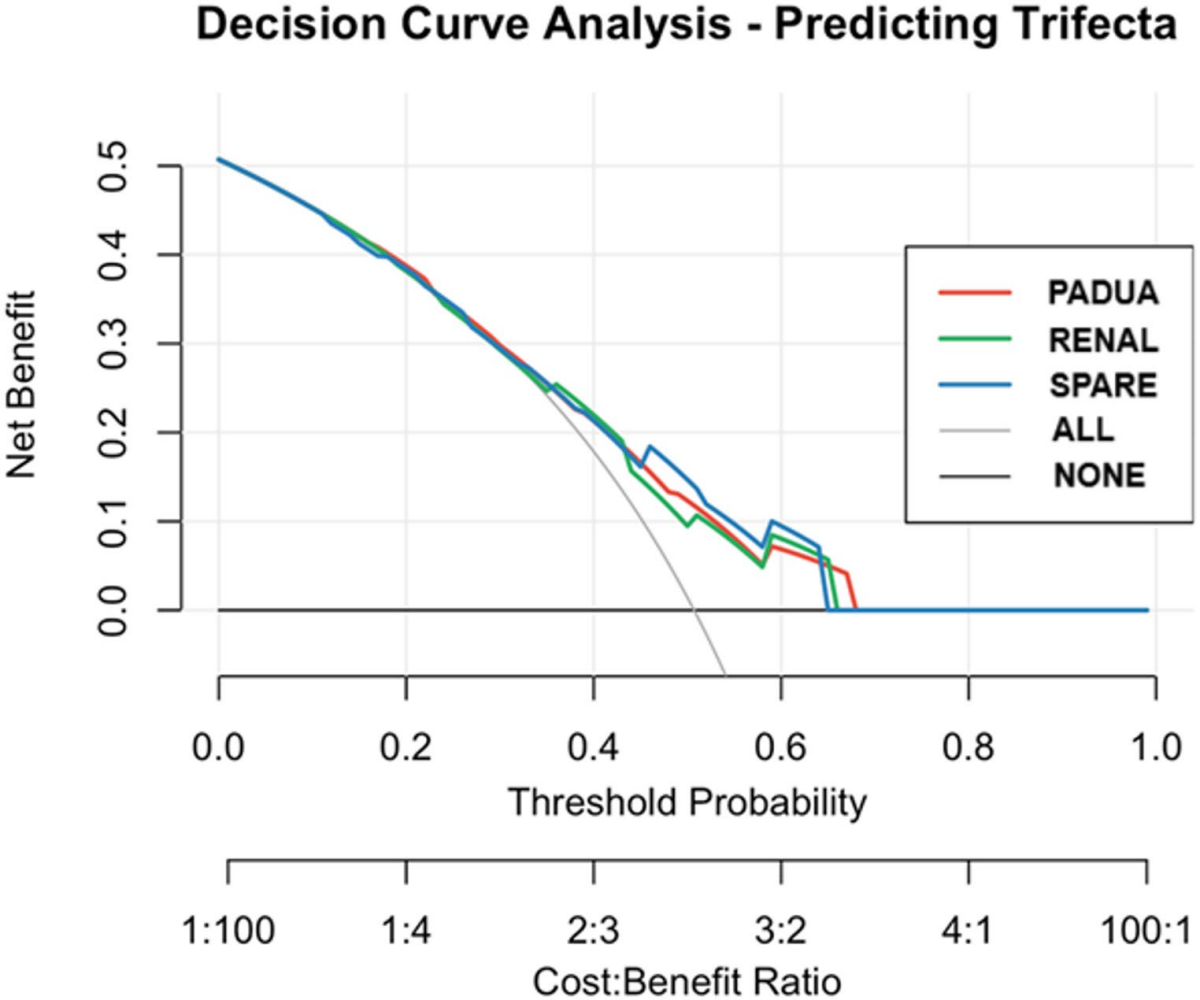
Decision curve analysis predicting Trifecta

DCA for Trifecta achievement (Fig. 2) demonstrated a substantial overlap across the three models, indicating similar clinical utility. With small net benefit differences in the mid thresholds (0.25–0.45), the SPARE score performed marginally better compared to the PADUA and RENAL scores, suggesting a marginal advantage.

## Discussion

In our study, we performed an external validation and comparative analysis of the PADUA, RENAL, and SPARE scores to predict Trifecta achievement in the context of SP-RAPN. Our findings show that all three scores demonstrated fair discriminative ability, with the SPARE score reaching the highest AUC (0.681), followed by the PADUA (0.661) and the RENAL (0.654) scores. However, none of the differences between models reached statistical significance based on DeLong’s tests. These results suggest that SPARE, PADUA, and RENAL provide comparable predictive value for the Trifecta outcome in the SP-RAPN setting. To the best of our knowledge, this is the first external validation study specifically addressing the performance of nephrometry scores in SP-RAPN.

Our findings are consistent with previous external validation of the three scores in multiport robotic cohorts. Khene et al. [14] demonstrated that while PADUA, RENAL, and SPARE were all significantly associated with overall complications, their discriminative ability was modest and comparable to tumor size alone. Similarly Veccia et al. [4] reported no significant advantage of the SPARE score over the PADUA or RENAL score in predicting complications. Notably, multiple studies support the SPARE score as a practical and reproducible alternative to the more complex PADUA and RENAL scores. Huang et al. [17] found that SPARE had comparable predictive ability to PADUA and RENAL for predicting composite outcomes. Crockett et al. [18], in the only study specifically addressing retroperitoneal multiport RAPN, reported that only the SPARE and PADUA, but not the RENAL score, were able to predict Trifecta. While these results align with our finding that SPARE achieved a better performance for Trifecta prediction, the differences between the three scores were not statistically significant. Interestingly, tumors with endophytic growth or T1b were consistently associated with lower Trifecta achievement, reinforcing the impact of anatomical complexity on surgical outcomes. This association is particularly relevant in retroperitoneal approaches, where reduced working space and challenging angles may increase technical difficulty.

When analyzing Trifecta outcomes, it is important to contextualize our findings within existing literature. In our series, the overall Trifecta achievement rate was slightly lower than that typically reported for multi-port RAPN [15]. This difference likely reflects variability in the definition of Trifecta applied across studies. The definition by Khalifeh et al. included the simultaneous achievement of negative surgical margins, absence of perioperative complications, and warm ischemia time < 25 min [15]. Others studies have proposed modified criteria, such as incorporating functional endpoints like postoperative Estimated Glomerular Filtration Rate (eGFR) decline < 10% [19], or adopting definitions based on complication severity, such as Clavien–Dindo grade < III with eGFR decline < 30% [20], or Clavien Dindo grade < II with no postoperative acute kidney injury [21]. Prior comparisons have shown that SP-RAPN achieves perioperative outcomes and oncologic efficacy comparable to those of MP- RAPN [22], supporting that any discrepancies in composite outcomes largely depend on definitional variability rather than true performance differences. The predictive value of each score appears closely tied to the technical characteristics and limitations of the surgical approach. As novel multiport robotic systems continue to evolve [23], so too must our evaluation of their clinical impact. In this context, even the variability in access route, such as lateral flank versus low anterior, can influence surgical outcomes [24].

To date, no nephrometry score has been specifically developed considering technical aspects such as hilar exposure, limited working angle, or perinephric fat distribution, factors particularly relevant in retroperitoneal SP-RAPN. The only exception is the Retroperitoneal Nephrometry Scoring System [25], which was developed specifically for retroperitoneal minimally invasive partial nephrectomy but has not been evaluated in SP cohorts. Although not assessed in our analysis, its development highlights the need for tailored nephrometric tools to optimize patient selection and procedural planning in SP-RAPN.

These observations have practical implications for the growing field of SP-RAPN. First, if confirmed with larger, multi-institutional SP cohorts, the slightly superior, but not statistically significant, predictive ability of SPARE for Trifecta may support its adoption as the preferred and reference standard score for reporting tumor complexity and surgical outcomes in clinical practice. Second, the absence of a SP-specific nephrometry system highlights an opportunity to develop new tools tailored to the technical demands of retroperitoneal SP-RAPN.

Our study is strengthened by several factors: its bi-institutional design involving two experienced centers for SP robotic surgery, the blinded scoring of nephrometry systems and the use of a clinically meaningful composite endpoint, which provides a comprehensive assessment of surgical success.

Several limitations should be acknowledged. First, the retrospective design introduces potential selection bias. Second, our analysis was limited to three main nephrometry scores, excluding alternative systems that may offer additional insights. Third, the six-month follow-up, while adequate for assessing perioperative outcomes and composite achievement, does not capture long-term renal function or oncologic recurrence. Lastly, highly complex tumors were underrepresented, which may limit the applicability of our findings to the most challenging surgical cases.

Looking ahead, the integration of anatomical scoring systems with immersive technologies including virtual reality platforms [26] within metaverse environments [27–29] will play a central role in the future of surgical planning. When combined with AI-driven data analysis and simulation, these tools may enable surgeons to refine preoperative strategies, enhance intraoperative navigation, and ultimately improve clinical outcomes, as recently demonstrated [30] during augmented reality–guided RAPN.

## Conclusions

SPARE, PADUA and RENAL scores have comparable performance in predicting Trifecta in the context of SP-RAPN. SPARE shows a slightly higher discriminative ability, but without reaching statistically significant difference. Given its simplicity and ease of use, SPARE may represent a practical option for standardized preoperative assessment. Future studies are needed to validate these findings in larger SP-RAPN.

## Supplementary Information

Below is the link to the electronic supplementary material.

Supplementary Material 1

## Data Availability

No datasets were generated or analysed during the current study.
