# Supplementary material for: Predicting surgical outcomes in single-port robot-assisted partial nephrectomy: external validation and comparative analysis of PADUA, RENAL, and SPARE scores
Source: World J Urol. 2025 Nov 20;43(1):707. doi: 10.1007/s00345-025-06081-7 (PMC12634722; doi:10.1007/s00345-025-06081-7)
Supplement: Supplementary file 1 — Supplementary Material 1 [file 345_2025_6081_MOESM1_ESM.docx]

**Supplementary Table 1. RENAL, PADUA, and SPARE scores**

| Variables | RENAL score | PADUA score | SPARE score |
| --- | --- | --- | --- |
| Polar location | Not included |  | Not included |
| Upper/lower |  | 1 |  |
| Medium |  | 2 |  |
| Exophytic rate |  |  |  |
| ≥50% | 1 | 1 | 0 |
| <50% | 2 | 2 | 1 |
| Endophytic | 3 | 3 | 2 |
| Rim location | Not included |  |  |
| Lateral |  | 1 | 0 |
| Medial |  | 2 | 2 |
| Renal sinus involvement | Not included |  |  |
| Absent |  | 1 | 0 |
| Present |  | 2 | 3 |
| Urinary collecting system involvement | Not included |  | Not included |
| Absent |  | 1 |  |
| Present |  | 2 |  |
| Maximum tumor size, (cm) |  |  |  |
| ≤4 | 1 | 1 | 0 |
| 4,1-7 | 2 | 2 | 2 |
| >7 | 3 (≥ 7) | 3 | 4 |
| Nearness to the collecting system or sinus, (mm) |  | Not included | Not included |
| ≤4 | 1 |  |  |
| 4,1-7 | 2 |  |  |
| ≥7 | 3 |  |  |
| Location relative to the polar lines: |  | Not included | Not included |
| Entirely above the upper or below the lower polar line | 1 |  |  |
| Crosses polar line | 2 |  |  |
| >50% is across polar line or crosses the axial renal midline or is entirely between the polar lines | 3 |  |  |

Abbreviations: PADUA = Preoperative Aspects and Dimensions Used for an Anatomical classification; RENAL = Radius, Exophytic/Endophytic, Nearness, Anterior/Posterior, Location; SPARE = Simplified PADUA REnal nephrometry score.

**Supplementary Table 2. Components of scores**

| **Variables** | |  |
| --- | --- | --- |
| **PADUA** | **Polar location, n (%)** |  |
|  | Upper/lower | 139 (65.9) |
|  | Medium | 72 (34.1) |
|  | **Exophytic rate, n (%)** |  |
|  | ≥50% | 103 (48.8) |
|  | <50% | 85 (40.3) |
|  | Endophytic | 23 (10.9) |
|  | **Rim location, n (%)** |  |
|  | Lateral | 138 (65.4) |
|  | Medial | 73 (34.6) |
|  | **Renal sinus involvement, n (%)** |  |
|  | Absent | 178 (84.4) |
|  | Present | 33 (15.6) |
|  | **Urinary collecting system involvement, n (%)** |  |
|  | Absent | 183 (86.7) |
|  | Present | 28 (13.3) |
|  | **Maximum tumor size, cm, n (%)** |  |
|  | ≤4 | 166 (78.7) |
|  | 4.1-7 | 39 (18.5) |
|  | >7 | 6 (2.8) |
| **SPARE** | **Rim location, n (%)** |  |
|  | Lateral | 138 (65.4) |
|  | Medial | 73 (34.6) |
|  | **Renal sinus involvement, n (%)** |  |
|  | Absent | 178 (84.4) |
|  | Present | 33 (15.6) |
|  | **Exophytic rate, n (%)** |  |
|  | ≥50% | 103 (48.8) |
|  | <50% | 85 (40.3) |
|  | Endophytic | 23 (10.9) |
|  | **Maximum tumor size, cm, n (%)** |  |
|  | ≤4 | 166 (78.7) |
|  | 4.1-7 | 39 (18.5) |
|  | >7 | 6 (2.8) |
| **RENAL** | **Radius, cm, n (%)** |  |
|  | ≤4 | 166 (78.7) |
|  | 4.1-7 | 39 (18.5) |
|  | ≥7 | 6 (2.8) |
|  | **Renal exophytic, n (%)** |  |
|  | ≥50% | 103 (48.8) |
|  | <50% | 85 (40.3) |
|  | Entirely endophytic | 23 (10.9) |
|  | **Nearness to collecting system or sinus, mm, n (%)** |  |
|  | ≥7 | 138 (65.4) |
|  | 4-7 | 20 (9.5) |
|  | ≤4 | 53 (25.1) |
|  | **Location, n (%)** |  |
|  | Upper polar line | 122 (57.8) |
|  | Crosses polar line | 51(24.2) |
|  | Between polar line | 38(18.0) |
|  | **Hilar, n (%)** |  |
|  | Yes | 201(95.3) |
|  | No | 10(4.7) |

Abbreviations: PADUA = Preoperative Aspects and Dimensions Used for an Anatomical classification; RENAL = Radius, Exophytic/Endophytic, Nearness, Anterior/Posterior, Location; SPARE = Simplified PADUA REnal nephrometry score.

|  | | Logistic Regression | | | |
| --- | --- | --- | --- | --- | --- |
|  |  | Univariate | | Multivariate | |
| Variables | | OR (95%CI) | P value | OR (95%CI) | P value |
| **PADUA** | **Polar location:**  Middle vs superior/inferior | 0.958 (0.541-1.694) | 0.882 | 1.141 (0.601-2.186) | 0.688 |
|  | **Exophytic rate:**  <50% vs ≥50%  Endophytic vs <50% | 0.322 (0.175-0.581)  0.431 (0.168-1.074) | **<0.001**  0.072 | 0.346 (0.179-0.658)  0.480 (0.171-1.332) | **0.001**  0.158 |
|  | **Rim location:**  Medial vs lateral | 0.465 (0.258-0.826) | **0.009** | 0.569 (0.292-1.104) | 0.096 |
|  | **Sinus involvement:**  Involved vs uninvolved | 0.427 (0.189-0.916) | **0.033** | 0.836 (0.292-2.349) | 0.734 |
|  | **Urinary collecting system:**  Dislocated/involved vs not | 0.695 (0.305-1.543) | 0.374 | 1.254 (0.452-3.574) | 0.665 |
|  | **Size group:**  4.1-7cm vs ≤4cm  >7cm vs ≤4cm | 0.235 (0.107-0.483)  0.726 (0.028-18.579) | **<0.001**  0.822 | 0.237 (0.104-0.510)  1.425 (0.053-39.090) | **<0.001**  0.809 |
| **SPARE** | **Rim location:**  Medial vs lateral | 0.507 (0.283-0.899) | **0.021** | 0.655 (0.334-1.282) | 0.217 |
|  | **Sinus involvement:**  Involved vs not involved | 0.127 (0.189-0.916) | **0.033** | 0.911 (0.351-2.322) | 0.845 |
|  | **Exophytic rate:**  <50% vs ≥50%  Endophytic vs ≥50% | 0.322 (0.175-0.581)  0.431 (0.168-1.074) | **<0.001**  0.072 | 0.345 (0.178-0.659)  0.553 (0.206-1.484) | **0.001**  0.236 |
|  | **Size group:**  4.1-7cm vs ≤4cm  >7cm vs ≤4cm | 0.235 (0.107-0.483)  0.726 (0.028-18.579) | **<0.001**  0.822 | 0.229 (0.099-0.491)  1.446 (0.054-38.851) | **<0.001**  0.800 |
| **RENAL** | **Radius:**  4-7cm vs ≤4cm  >7cm vs ≤4cm | 0.235 (0.107-0.483)  0.726 (0.028-18.579) | **<0.001**  0.822 | 0.266 (0.115-0.583)  1.589 (0.059-42.757) | **0.001**  0.751 |
|  | **Renal exophytic:**  <50% vs ≥50%  Endophytic vs ≥50% | 0.322 (0.175-0.581)  0.431 (0.168-1.074) | **<0.001**  0.072 | 0.331 (0.172-0.627)  0.506 (0.179-1.415) | **<0.001**  0.191 |
|  | **Nearness collecting system/sinus:**  4.1-7mm vs <4mm  >7mm vs <4mm | 0.913 (0.355-2.399)  0.353 (0.177-0.679) | 0.849  **0.002** | 1.269 (0.458-3.628)  0.584 (0.257-1.305) | 0.649  0.191 |
|  | **Location:**  Crosses polar line vs entirely above/below polar lines  Between polar line vs entirely above/below polar lines | 0.779 (0.403-1.499)    0.789 (0.378-1.638) | 0.456  0.525 | 1.189 (0.558-2.567)  1.133 (0.490-2.661) | 0.655  0.772 |
|  | **Hilar:**  Yes vs no | 0.634 (0.158-2.287) | 0.491 | 0.998 (0.217-4.279) | 0.998 |

**Supplementary Table 3. Score components analysis of Trifecta achievement**

Abbreviations: OR = Odds Ratio; CI = Confidence Interval; PADUA = Preoperative Aspects and Dimensions Used for an Anatomical classification; RENAL = Radius, Exophytic/Endophytic, Nearness, Anterior/Posterior, Location; SPARE = Simplified PADUA REnal nephrometry score.
